# Early prediction of in-hospital deterioration after emergency department admission using machine learning models

**DOI:** 10.1186/s12873-025-01464-w

**Published:** 2026-01-05

**Authors:** Chi-Yung Cheng, Ting-Hsuan Hsu, Yu-Lun Hung, Ting-Yu Hsu, Fu-Jen Cheng, Hsiu-Yung Pan, Chun-Hung Richard Lin, I-Min Chiu

**Affiliations:** 1 Department of Emergency Medicine, Kaohsiung Chang Gung Memorial Hospital, Chang Gung University College of Medicine, No. 123, Ta Pei Road, Niao Sung Hsiang, Kaohsiung City, 833 Taiwan; 2 Department of Emergency Medicine, Kaohsiung Municipal Feng Shan Hospital Under The Management of Chang Gung Medical Foundation, Kaohsiung, Taiwan; 3 Department of Computer Science and Engineering, National Sun Yat-sen University, Kaohsiung, Taiwan

**Keywords:** Machine learning, Early warning system, In-hospital deterioration

## Abstract

**Background:**

Early prediction of clinical deterioration in patients admitted to general wards from the emergency department (ED) is crucial for timely interventions and improved outcomes. Traditional scoring systems often fail to account for dynamic physiological changes occurring during ED stays. Machine learning (ML) offers a promising alternative by integrating comprehensive patient data for enhanced predictive capabilities.

**Objective:**

This study aimed to develop and validate an ML-based early warning system to predict adverse events, including cardiac arrest, mechanical ventilation, or intensive care unit (ICU) transfer, within 48 h of hospitalization.

**Methods:**

This retrospective multicenter study included data from 169,807 patients across two medical centers to train ML models for predicting adverse events occurring within 48 h of hospitalization. The prediction time origin (T0) was the moment of hospital admission. All ED data obtained before T0 were included as model input, and adverse events were defined as those occurring within 48 h after T0. Three machine learning models were trained and evaluated: logistic regression (LR), random forest (RF), and extreme gradient boosting (XGB). The ML models were evaluated in external test cohorts with 54,515 patients from a regional teaching hospital. Model performance was assessed against traditional early warning scores, including the National Early Warning Score (NEWS) and the Modified Early Warning Score (MEWS), and Modified Sequential Organ Failure Assessment Score (mSOFA) using the area under the curve (AUC).

**Results:**

In the internal test set, XGB achieved the highest AUC of 0.87 (95% CI: 0.85–0.89), followed by RF 0.85 (95% CI: 0.83–0.87) and LR 0.83 (95% CI: 0.81–0.85), outperforming NEWS (AUC 0.71), MEWS (0.69), and mSOFA (0.69). In the external test set, XGB maintained the best discrimination (AUC 0.82, 95% CI: 0.80–0.83), followed by RF 0.78 (95% CI: 0.77–0.80) and LR 0.77 (95% CI: 0.76–0.79), again exceeding NEWS (0.67), MEWS (0.65), and mSOFA (0.69). SHAP analyses identified respiratory rate and oxygen support as the most influential predictors.

**Conclusion:**

The ML-based early warning system demonstrated strong predictive performance across diverse healthcare settings, significantly surpassing the capabilities of traditional scoring systems. This approach has the potential to improve risk stratification and enable timely interventions, ultimately enhancing patient outcomes.

**Supplementary Information:**

The online version contains supplementary material available at 10.1186/s12873-025-01464-w.

## Introduction

Ensuring patient safety and improving the quality of care remain key priorities in emergency medicine. One of the ongoing challenges in emergency department (ED) is the early recognition of clinical deterioration, especially in patients who appear stable during their ED stay but later experience adverse events after transfer to general wards. This highlights the importance of identifying high-risk patients in a timely manner to facilitate early interventions and adjust disposition plan. Commonly proposed strategies include prolonged observation, early therapeutic interventions, and the use of risk stratification tools to assess and prioritize patients based on their likelihood of deterioration.

Various risk stratification systems have been developed to assist clinicians in evaluating patient conditions. However, many commonly used tools, such as the Acute Physiology and Chronic Health Evaluation (APACHE) [1, 2], Simplified Acute Physiology Score (SAPS) [3, 4], and Laboratory-based Acute Physiology Score (LAPS) [5], were initially designed for critically ill patients in intensive care units (ICUs). When applied to intermediate risk patients that required ward admission after initial resuscitation, these tools may introduce biases or fail to accurately predict early deterioration [6–8]. Most rapid risk classification tools, such as the National Early Warning Score (NEWS) and Modified Early Warning Score (MEWS) [9, 10], rely predominantly on single-point vital sign measurements taken at a specific time. They do not account for serial measurements, laboratory results, or other clinical data gathered during the entirety of the ED stay, limiting their ability to reflect the dynamic and evolving nature of patient conditions.

Artificial intelligence has emerged as a transformative tool in healthcare, offering advancements in clinical diagnosis, treatment planning, and the prediction of health outcomes and mortality [11–14]. Several machine learning (ML) based models have been proposed for ED risk classification, addressing tasks such as sepsis detection, mortality prediction, and triage optimization [15–19]. For instance, a study developed an ML-based real-time prediction model for short-term mortality in critically ill patients, which outperformed conventional risk scores like the NEWS in both internal and external validations [20]. Another research effort created an interpretable ML model to predict in-hospital mortality among sepsis patients, demonstrating superior performance compared to traditional severity scores such as the Sequential Organ Failure Assessment (SOFA) [21]. Although these systems provide valuable insights, they are limited in their ability to predict early deterioration among patients admitted to general wards from the ED.

Unplanned intensive care unit (ICU) transfer, mechanical ventilation initiation, or cardiac arrest shortly after ward admission are commonly considered markers of disposition error, in which a patient is under-triaged to a lower level of care, resulting in delayed escalation and potentially worse outcomes. Prior studies have demonstrated that patients requiring ICU transfer within 24 h of admission experience significantly increased mortality and length of stay [22–25]. In this study, we hypothesize a ML-based solution can predict early inhospital deterioration at the check point before patient’s admission. We aimed to develop and validate a ML-based early warning system to predict adverse events in patients admitted to general wards from the ED, leveraging data from a multicenter cohort to address the limitations of existing tools and improve clinical outcomes.

## Method

### Study setting and population

**Fig. 1:**
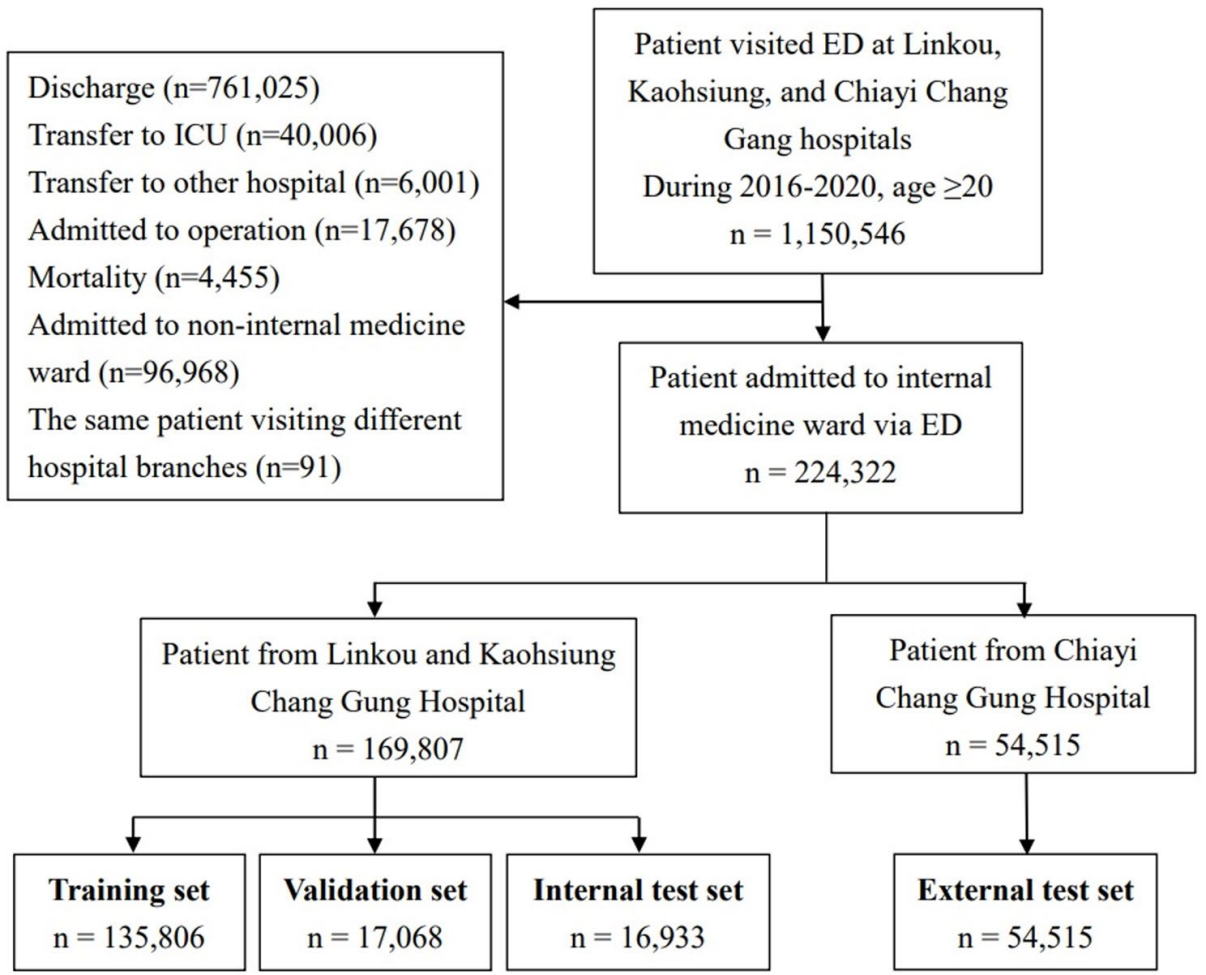
Patient inclusion flow chart

The study collected electronic health records from two medical centers, Linkou and Kaohsiung Chang Gung Memorial Hospitals (CGMH) in northern and southern Taiwan, to be the development cohort. Data collected from January 1, 2016, to December 31, 2020, were analyzed. The study included non-traumatic patients aged over 20 who were admitted to hospital wards through the ED. Patients transferred directly to the ICU from the ED, discharged against medical advice, or transferred to other hospitals during their admission were excluded from the analysis. The ML model was external validated in a regional teaching hospital, Chiayi CGMH. This study was conducted in accordance with the Declaration of Helsinki and was approved by the Institutional Review Board of the Chang Gung Medical Foundation. Due to the retrospective design, informed consent was not required. The study flow chart in Fig. 1 illustrates this selection process.

### Data collection and preprocessing

The study gathered a comprehensive range of variables to analyze key prognostic factors, focusing on patients’ medical history, vital signs, laboratory results, and medical treatment during ED visits. Demographic data, such as age and sex, were included, along with vital signs measured at two key time points: one at ED triage and another at the time before transfering to hospital wards. These dual measurements aimed to capture any changes in the patient’s condition during their ED stay. The recorded vital signs included heart rate (HR), systolic blood pressure (SBP), diastolic blood pressure (DBP), body temperature (BT), respiratory rate (RR), and Glasgow Coma Scale (GCS) scores, encompassing the eye response (GCSE), verbal response (GCSV), and motor response (GCSM) components. Additionally, the shock index (SI), defined as heart rate divided by systolic blood pressure, was used as a monitoring metric to assess clinical status [26].

Laboratory results, including complete blood counts, differential white blood cell counts, C-reactive protein levels, renal function, liver enzymes, and electrolytes, were also analyzed. The patients’ underlying medical history were also collected as input variables, including hypertension, diabetes mellitus (DM), liver cirrhosis, cerebrovascular accident (CVA), heart failure, coronary artery disease (CAD), end renal stage disease (ESRD), and malignancy,

Management details in the ED, including both pharmacological and non-pharmacological interventions, were incorporated as critical variables. This included fluid resuscitation, oxygen therapy, and the use of inotropic agents. Oxygen support was defined as the administration of supplemental oxygen via conventional low-flow delivery devices. Patients receiving high-flow oxygen support were also categorized as receiving oxygen support and included within this variable to reflect overall oxygen supplementation. To differentiate the timing of measurements, suffixes were applied to the variables: “_a” for those collected during ED triage and “_e” for those measured upon transfer to the ward (e.g., BT_a for body temperature at triage and BT_e for body temperature at ward admission).

To address missing data, we adopted an imputation strategy combined with explicit missingness indicators. For variables with missing values, multiple imputation was performed using the MissForest algorithm, which applies random forest models to iteratively predict missing entries based on observed data across other variables [27]. This approach allows for flexible modeling of nonlinear relationships and interactions among predictors and has demonstrated strong performance in handling mixed-type clinical data. In addition, for each variable with missing values, a corresponding binary missingness indicator was created to denote whether the original value was missing. These indicators were incorporated into model development to account for informative missingness, acknowledging that the absence of certain laboratory tests or measurements may itself carry clinical significance. All imputation procedures were performed using training data only, and the fitted imputation models were subsequently applied to the validation and test sets to prevent information leakage.

For each patient, the prediction time origin (T0) was defined as the moment of hospital admission, corresponding to ED disposition and bed assignment. All data elements used as predictors were restricted to those available at or prior to T0. No data collected after T0 were used for training or testing. The target outcome, early in-hospital deterioration, included cardiac arrest, requirement for mechanical ventilation, or transfer to the ICU.

To standardize the features for model training and evaluation, we applied the StandardScaler method. This approach transforms each feature by subtracting the mean and scaling to unit variance. The scaler was first fitted to the training dataset, and the learned parameters were subsequently applied to the validation set, internal test set, and external test set. This standardization ensured consistency across all datasets, preventing biases from differences in feature scaling.

### Model development and validation

During the model development phase, data from Linkou and Kaohsiung CGMH were divided into training, validation, and internal test sets at an 8:1:1 ratio., as illustrated in Fig. 1. All divisions were performed at the patient level to ensure that data from the same individual were confined to a single subset, thus preventing information leakage. This approach maintained distinct, non-overlapping datasets, providing a fair and unbiased framework for model training and assessment.

We trained and validated three ML algorithms in predicting early inhospital deterioration, including logistic regression (LR), random forest (RF), and extreme gradient boosting (XGB). LR is a supervised classification algorithm that applies a sigmoid function to its output to produce a probability, which can then be mapped to two or more discrete classes [28]. To optimize feature selection for the LR model, we applied a stepwise selection process based on the area under the precision-recall curve (AUC). Starting with an empty feature set, we iteratively added the feature that yielded the greatest AUC improvement on the validation set. The process stopped after three consecutive iterations without improvement to avoid overfitting. This approach efficiently identified the most predictive features while reducing redundancy. The final feature set was used to train the LR model for further evaluation.

RF and XGB are tree-based ensemble learning methods that are particularly suitable for high-dimensional clinical data [29, 30]. RF reduces overfitting through bootstrapped sampling and random feature selection at each split, while XGB employs gradient boosting with regularization to model complex nonlinear relationships and interactions among variables. Importantly, both algorithms perform implicit feature selection during training by prioritizing informative predictors and down-weighting less relevant or redundant features. Therefore, RF and XGB were trained using the full set of available variables without explicit feature preselection, allowing the models to leverage potentially informative clinical features while avoiding premature exclusion of relevant predictors. For RF and XGB, hyperparameter tuning was conducted via grid search, and the optimal configurations were selected based on validation set performance (AUC). The final models were then retrained on the full training data using these optimized hyperparameters and evaluated on the internal and external test sets without further adjustment.

### Model performance and statistical analysis

Continuous variables were described as the mean ± standard deviation (SD) for normally distributed data and as the median with interquartile ranges (IQR, 25th–75th percentiles) for non-normally distributed data. To evaluate the performance of the predictive model, we compared it with conventional early warning scores, including NEWS and MEWS, and modified Sequential Organ Failure Assessment score (mSOFA) [31]– [32]. NEWS and MEWS scores were calculated using vital signs measured at the prediction time origin T₀. The model’s predictive performance was measured using AUC. Group comparisons for continuous variables were performed using the independent t-test for normally distributed data or the Mann–Whitney U test for non-normally distributed data. Categorical variables were compared using the chi-square test for independence. Statistical significance was defined as a two-sided p-value < 0.01. Additionally, Shapley Additive Explanation (SHAP) was applied to interpret the contribution of each feature to the model’s predictions [33]. All analyses were performed using Python version 3.12 with the PyCharm integrated development environment (IDE) on a macOS platform.

## Result

### Analysis of the enrolled patients

A total of 169,807 patients from Linkou and Kaohsiung CGMH were divided into training (*n* = 135,806), validation (*n* = 17,068), and internal test (*n* = 16,933) sets. Patients from Chiayi CGMH were assigned to the external test set (*n* = 54,515). The mean age was highest in the external test set (68.9 ± 16.3 years, *p* < 0.001). Adverse events, including cardiac arrest, mechanical ventilation, and ICU transfer, were significantly less frequent in the external test set (1.5%, *p* < 0.001). Other baseline characteristics, ED management details, and outcomes are summarized in Table 1.

**Table 1:** Characteristics of patients in training, validation, internal test, and external test data set

	Training set *n* = 135,806	Validation set *n* = 17,068	Internal test set *n* = 16,933	External test set *n* = 54,515	*p*-value
Age, year-old, mean$\:\pm\:$SD	66.1 ± 16.3	66.3 ± 16.3	65.9 ± 16.4	68.9 ± 16.3	< 0.001
Male, n (%)	77,899 (57.4%)	9767 (57.2%)	9830 (58.1%)	31,014 (56.9%)	0.049
Body mass index	23.8 ± 4.0	23.7 ± 4.0	23.7 ± 3.9	23.7 ± 2.6	< 0.001
ED LOS, minutes	1926.7 ± 1725.9	1929.8 ± 1740.0	1912.5 ± 1719.4	901.6 ± 883.1	< 0.001
Vital sign at triage, mean$\:\pm\:$SD					
Temperature, °C	36.9 ± 1.1	37.0 ± 1.1	36.9 ± 1.1	36.9 ± 1.1	0.242
Heart rate, bpm	95.9 ± 21.8	95.6 ± 21.6	95.8 ± 21.7	95.7 ± 21.1	0.246
Systolic BP, mmHg	140.1 ± 32.4	140.4 ± 32.5	139.6 ± 32.4	137.3 ± 31.1	< 0.001
Diastolic BP, mmHg	80.0 ± 17.7	79.9 ± 17.9	80.1 ± 17.7	80.8 ± 16.9	< 0.001
Respiratory rate	19.6 ± 3.3	19.6 ± 3.3	19.7 ± 3.3	20.0 ± 2.7	< 0.001
Shock index	0.7 ± 0.3	0.7 ± 0.3	0.7 ± 0.3	0.7 ± 0.3	< 0.001
GCS, eye response	3.9 ± 0.5	3.9 ± 0.5	3.9 ± 0.5	3.9 ± 0.6	< 0.001
GCS, verbal response	4.5 ± 1.2	4.5 ± 1.2	4.5 ± 1.2	4.5 ± 1.2	0.889
GCS, motor response	5.7 ± 0.8	5.7 ± 0.8	5.7 ± 0.8	5.8 ± 0.7	< 0.001
Vital sign at admission, mean$\:\pm\:$SD					
Temperature, °C	36.4 ± 0.7	36.4 ± 0.7	36.4 ± 0.7	36.4 ± 0.7	< 0.001
Heart rate, bpm	85.2 ± 17.4	85.0 ± 17.4	85.1 ± 17.4	84.3 ± 16.6	< 0.001
Systolic BP, mmHg	130.4 ± 28.4	130.6 ± 28.4	130.6 ± 28.5	128.0 ± 27.3	< 0.001
Diastolic BP, mmHg	77.8 ± 15.1	77.7 ± 15.0	77.9 ± 15.2	80.1 ± 15.2	< 0.001
Respiratory Rate	18.8 ± 2.7	18.8 ± 2.7	18.8 ± 2.6	19.8 ± 3.1	< 0.001
Shock index	0.7 ± 0.2	0.7 ± 0.2	0.7 ± 0.2	0.7 ± 0.2	< 0.001
GCS, eye response	3.9 ± 0.4	3.9 ± 0.4	3.9 ± 0.4	3.9 ± 0.5	< 0.001
GCS, verbal response	4.5 ± 1.1	4.5 ± 1.1	4.5 ± 1.1	4.5 ± 1.1	< 0.001
GCS, motor response	5.8 ± 0.7	5.8 ± 0.7	5.8 ± 0.7	5.8 ± 0.6	< 0.001
Underlying Medical History, N (%)					
Hypertension	57,568 (42.4%)	7234 (42.4%)	7099 (41.9%)	26,027 (47.7%)	< 0.001
Diabetes mellitus	41,852 (30.8%)	5324 (31.2%)	5159 (30.5%)	19,753 (36.2%)	< 0.001
Liver cirrhosis	13,636 (10.0%)	1665 (9.8%)	1795 (10.6%)	7079 (13.0%)	< 0.001
CVA	24,321 (17.9%)	3125 (18.3%)	3031 (17.9%)	8646 (15.9%)	< 0.001
Heart failure	16,690 (12.3%)	2111 (12.4%)	2065 (12.2%)	6673 (12.2%)	0.955
CAD	18,299 (13.5%)	2399 (14.1%)	2337 (13.8%)	7478 (13.7%)	0.109
ESRD	31,315 (23.1%)	3966 (23.2%)	3764 (22.2%)	12,710 (23.3%)	0.031
Malignancy	39,204 (28.9%)	4903 (28.7%)	4794 (28.3%)	15,041 (27.6%)	< 0.001
ED management					
Oxygen support	50,661 (37.3%)	6240 (36.6%)	6300 (37.2%)	17,821 (32.7%)	< 0.001
High-flow oxygen support	4256 (3.1%)	510 (3.0%)	525 (3.1%)	318 (0.6%)	< 0.001
Fluid challenge	2641 (1.9%)	314 (1.8%)	330 (1.9%)	318 (0.6%)	< 0.001
Inotropic	17,367 (12.8%)	2174 (12.7%)	2154 (12.7%)	8165 (15.0%)	< 0.001
Outcome, N (%)					
Adverse Event	3329 (2.5%)	400 (2.3%)	379 (2.2%)	814 (1.5%)	< 0.001
Cardiac Arrest	358 (0.3%)	30 (0.2%)	33 (0.2%)	53 (0.1%)	< 0.001
Mechanical Ventilation	2876 (2.1%)	351 (2.1%)	339 (2.0%)	653 (1.2%)	< 0.001
ICU transfer	2653 (2.0%)	329 (1.9%)	319 (1.8%)	624 (1.1%)	< 0.001

ED: emergency department, LOS: length of stay, BP: blood pressure, GCS: Glasgow coma scale, CVA: cerebrovascular accident, CAD: coronary artery disease, ESRD: end renal stage disease, ICU: intensive care unit

There were 4,922 (2.19%) patients with adverse events. The mean age was significantly higher in the adverse event group (71.3 ± 14.5 years vs. 66.7 ± 16.4 years, *p* < 0.001). Patients with adverse events exhibited significant differences in vital signs at both ED triage and admission, characterized by higher heart rates, respiratory rates, and shock index, alongside lower systolic and diastolic blood pressures (all *p* < 0.001). Laboratory findings showed elevated white blood cell counts, CRP, and creatinine levels, as well as reduced hemoglobin and albumin levels in the adverse group (all *p* < 0.001). Furthermore, patients with adverse events had higher prevalence rates of heart failure, coronary artery disease, and end-stage renal disease and were more likely to undergo intensive ED management, such as oxygen therapy, fluid challenges, and inotropic agent usage (all *p* < 0.001). Additional findings are summarized in Supplemental Table 1.

### Model performance

**Fig. 2:**
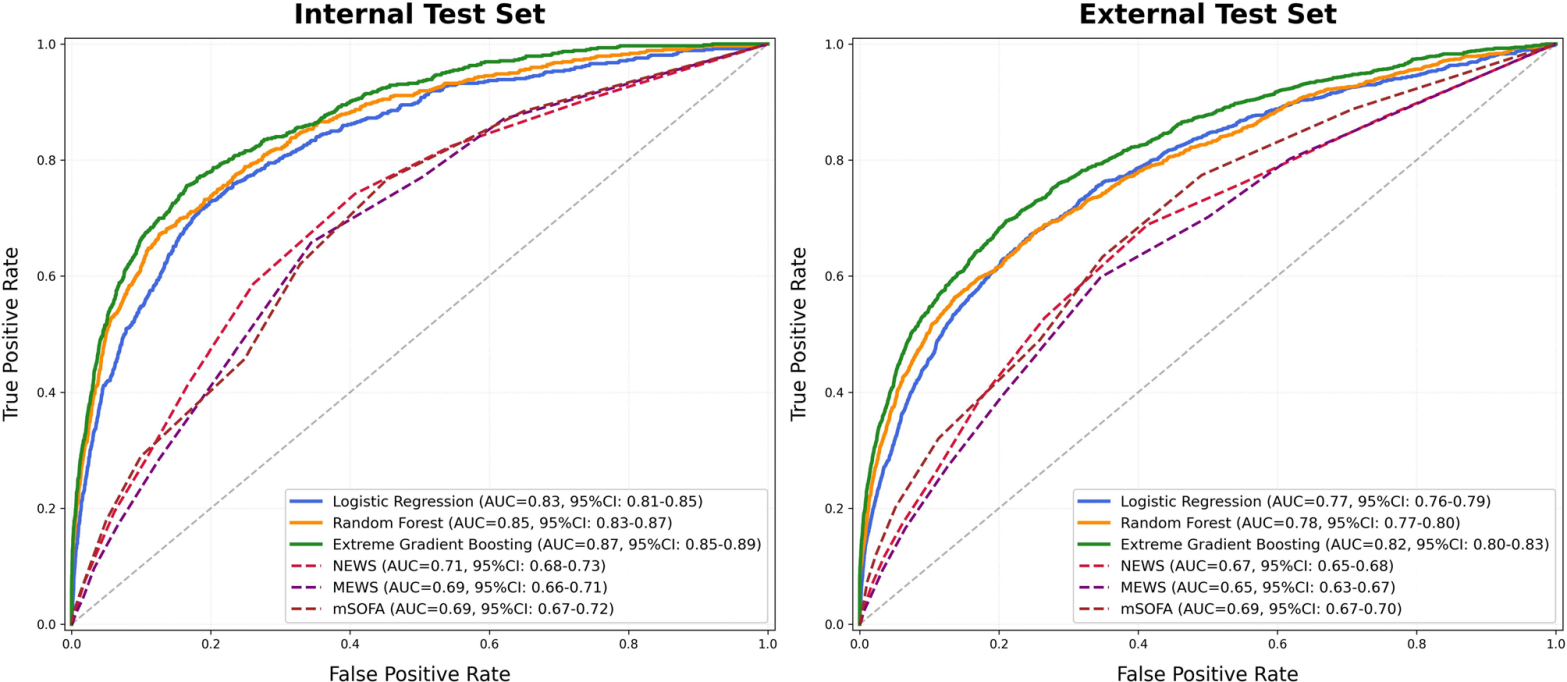
ROC curve of model prediction

As shown in Fig. 2, the ROC curves demonstrate that machine learning models consistently outperformed traditional early warning scores in both internal and external test sets. In the internal test set, XGB achieved the highest discrimination with an AUC of 0.87 (95% CI: 0.85–0.89), followed by random forest with an AUC of 0.85 (95% CI: 0.83–0.87) and logistic regression with an AUC of 0.83 (95% CI: 0.81–0.85). In contrast, conventional scoring systems showed substantially lower performance, with NEWS reaching an AUC of 0.71 (95% CI: 0.68–0.73), MEWS 0.69 (95% CI: 0.66–0.71), and mSOFA 0.69 (95% CI: 0.67–0.72). A similar pattern was observed in the external test set. XGB again demonstrated the best discrimination with an AUC of 0.82 (95% CI: 0.80–0.83), followed by random forest at 0.78 (95% CI: 0.77–0.80) and logistic regression at 0.77 (95% CI: 0.76–0.79). Traditional scores exhibited lower predictive accuracy, with NEWS at 0.67 (95% CI: 0.65–0.68), MEWS at 0.65 (95% CI: 0.63–0.67), and mSOFA at 0.69 (95% CI: 0.67–0.70).

**Fig. 3:**
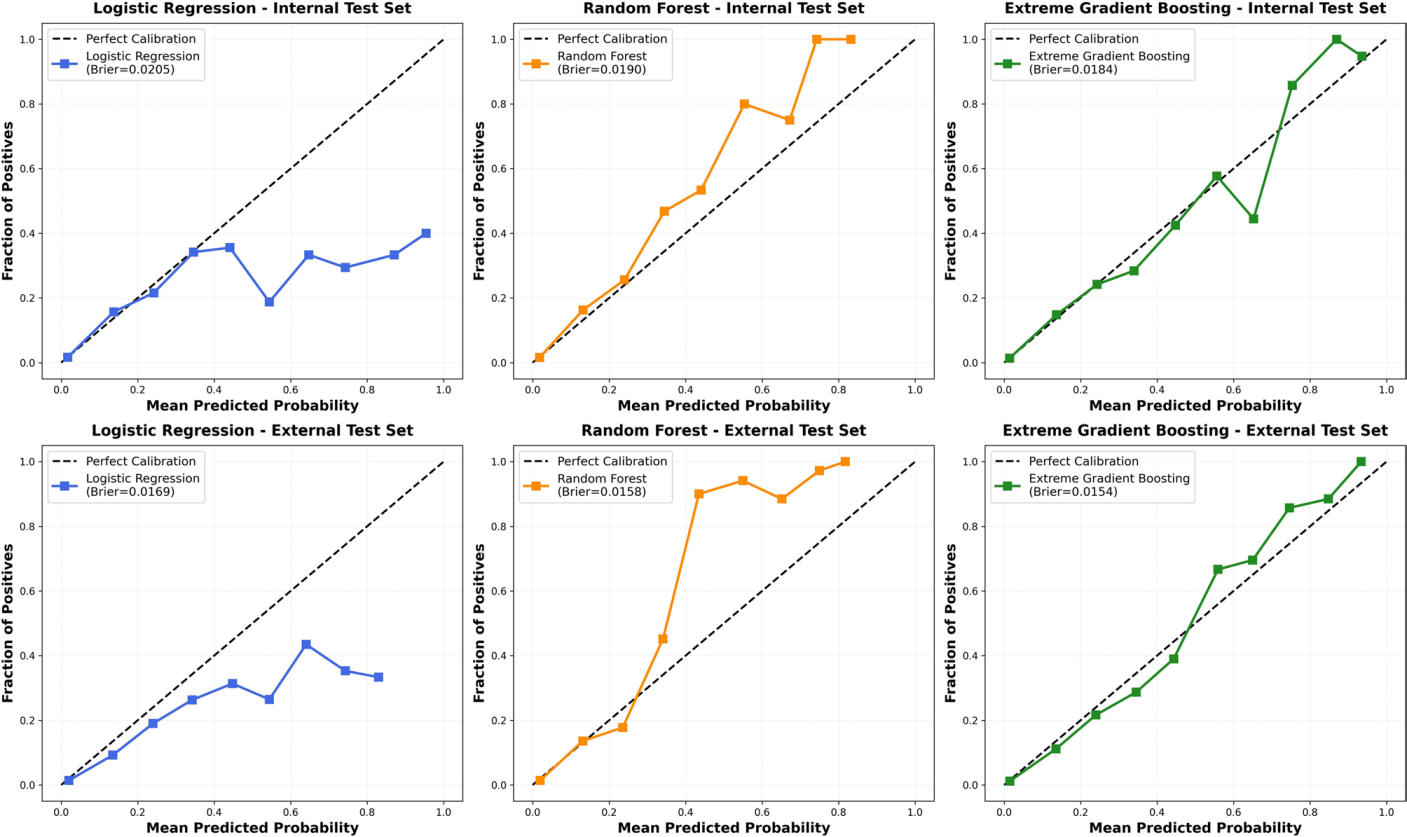
Calibration plots of the three machine learning models in the internal and external test sets

Figure 3 illustrates the calibration characteristics of the three machine learning models in both the internal and external test sets. LR showed a tendency to underestimate risk, with Brier scores of 0.0205 in the internal test set and 0.0169 in the external set. RF overestimated risk at higher predicted probabilities and had Brier scores of 0.0190 internally and 0.0158 externally. XGB demonstrated the most favorable calibration, with curves closely following the ideal diagonal and Brier scores of 0.0184 in the internal test set and 0.0154 in the external set.

**Fig. 4:**
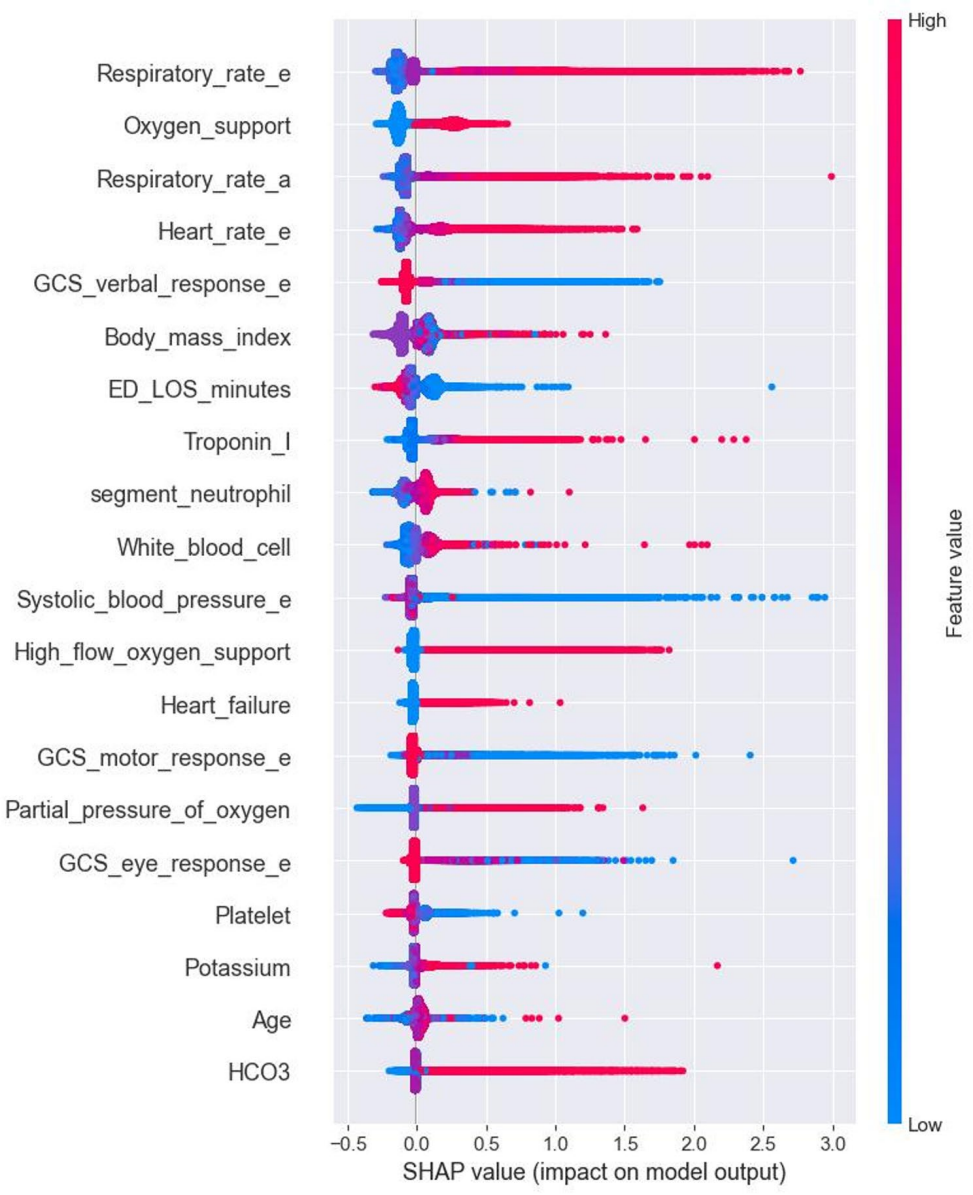
Global explanation of feature importance using Shapley Additive Explanation (SHAP) values for XGB

Figure 4 shows the SHAP summary plot illustrating the contribution of individual features to the predictions of the XGB model for early in-hospital deterioration. Features are ordered by their average absolute SHAP values, representing their importance in the model. The top predictors include RR_e, oxygen support, and RR_a, followed by HR_e and GCSV_e. These findings indicate that the model primarily relies on vital signs and oxygen requirements to identify patients at risk of deterioration.

**Fig. 5:**
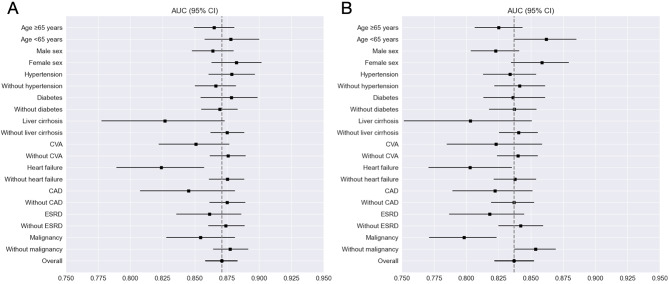
Subgroup analysis of XGB performance in predicting early in-hospital deterioration. (**A**) Internal test set, (**B**) External test set

Further evaluation of XGB performance was conducted across various patient subgroups, including older individuals and those with hypertension, DM, liver cirrhosis, prior stroke, heart failure, CAD, ESRD, and malignancy, as these groups are generally considered high-risk. The model’s AUC for predicting adverse events ranged from 0.778 to 0.902 in Linkou and Kaohsiung CGMH and from 0.767 to 0.885 in Chiayi CGMH across these subgroups. Detailed subgroup results are presented in Fig. 5, Supplemental Tables 3, and Supplemental Table 4.

## Discussion

The results showed that XGB consistently achieved higher predictive accuracy across the validation, internal, and external test sets compared to LR, RF, and traditional early warning scores. This underscores the ability of ML models to capture complex, non-linear relationships in clinical data, enabling more accurate predictions of adverse events. ML applications in risk classification have received significant attention for their ability to detect early signs of clinical deterioration and predict adverse outcomes. Lim et al. developed an ML-based model for real-time prediction of short-term mortality in critically ill ICU patients, achieving an impressive AUC of 0.87 in external validation and surpassing traditional scoring systems like NEWS [20]. Similarly, Li et al. created an interpretable ML model to predict in-hospital mortality among sepsis patients in the ICU by incorporating dynamic vital signs and laboratory data, achieving an AUC of 0.88 [34]. Our XGB model demonstrated similarly high AUC values in both internal and external test sets, confirming its effectiveness in identifying high-risk patients. However, unlike Lim et al. and Li et al., whose studies were confined to ICU populations, our research focuses on patients transitioning from the ED to general wards. This approach addresses the critical need for early warnings prior to ICU admission. Our findings support the concept that deterioration shortly after ward admission may reflect disposition error, in which patients who required higher acuity monitoring were initially placed in non-ICU settings. This phenomenon has been described in prior work examining unplanned ICU transfer and delayed escalation of care, and is associated with increased mortality and adverse outcomes. By predicting deterioration risk at T0, our model aims to reduce potentially preventable mis-dispositions and enable more appropriate initial bed placement.

For predicting acute clinical deterioration to support emergency care decision-making, Boulitsakis et al. compared multiple machine learning models, including logistic regression, gradient-boosted decision trees (GBDT), and support vector machines (SVM), with the National Early Warning Score 2 (NEWS2) for predicting mortality and critical care utilization within 24 h of admission [35]. Their study demonstrated that ML models, particularly GBDT, significantly outperformed NEWS2, achieving up to a 0.366 improvement in average precision and a 21.6% reduction in the daily alert rate. The study population consisted of unplanned emergency admissions across all specialties treated in a single institution’s Emergency Admissions Unit (EAU). In contrast, our study utilized a multicenter dataset, including two medical centers and a regional teaching hospital, capturing a broader spectrum of patient populations and clinical practices. This multicenter design enhances the generalizability of our findings, enabling the models to adapt effectively to diverse healthcare settings.

A previous systematic literature review provides a broad overview of various ML methods applied to predict patient deterioration, analyzing their performance across diverse clinical contexts, reporting a wide range of AUC values (0.55–0.99) depending on the methodology and context [36]. In our study, the external test set differed significantly from the other datasets, with a notably older patient population and higher prevalence of comorbidities such as hypertension, diabetes mellitus, liver cirrhosis, and malignancy. Despite this, the external test set had lower rates of interventions, including oxygen support and fluid challenges, and a significantly lower frequency of adverse events (1.5% vs. ~2.5%, *p* < 0.001). These differences may reflect the source of the data. While the training and internal validation datasets were derived from medical centers, the external test set came from a regional hospital. In clinical practice, more severe cases are less likely to be transported to regional hospitals by emergency medical technicians, as they often prioritize sending critically ill patients to medical centers with more advanced resources. Additionally, critically ill patients at the regional teaching hospital may have already been transferred to higher-level facilities, further reducing the number of severe cases included in the external test set. The ability of XGB and RF to maintain high AUC values in the external test set reflects their strength in handling heterogeneous datasets, capturing complex feature interactions, and generalizing across different clinical environments.

The SHAP summary plot indicates that the most influential features in the XGB model’s predictions for adverse events are RR_e, low oxygen support, and RR_a and HR_e. This suggests that respiratory status and hemodynamic stability are critical factors in anticipating patient deterioration. These findings are consistent with existing literature that highlights the importance of dynamic vital sign monitoring in predicting clinical deterioration. For instance, a study by Cheng et al. demonstrated that machine learning models analyzing vital sign dynamics could effectively predict in-hospital mortality in sepsis patients [16]. Similarly, Shamout et al. discussed the significance of continuous vital sign monitoring combined with machine learning algorithms in early detection of patient deterioration [37]. The application of SHAP values in this context provides transparent insights into the model’s decision-making process, enhancing interpretability and trust in the model’s predictions. This aligns with the growing emphasis on explainable AI in healthcare, as highlighted by recent studies advocating for the integration of interpretability methods like SHAP to understand model predictions better [38].

This study has several limitations. First, its retrospective design relies on the accuracy and completeness of electronic health records, which may introduce bias or unmeasured variability. Second, although we performed multicenter external validation, retrospective evaluation alone cannot fully reflect model performance in real-world clinical environments. Prior assessments of widely used clinical prediction systems such as the Rothman Index and the Epic Sepsis Model have shown that models with strong retrospective performance may experience substantial degradation when implemented prospectively, largely due to differences in case-mix, data quality, and clinical workflows [39–43]. These observations highlight the possibility that our model’s performance could also vary when deployed in routine practice. Future research should therefore include prospective validation and real-time evaluation to determine the model’s practical utility and impact on clinical decision-making. Broader evaluation across more diverse and heterogeneous populations will also be essential to ensure robustness and generalizability.

## Conclusion

This study developed and validated a machine learning-based early warning system to predict adverse events within 48 h among patients admitted to general wards from the ED. Using a multicenter cohort, we demonstrated the superior performance of machine learning models over traditional early warning scores such as the NEWS, MEWS, and mSOFA. These findings highlight the potential of ML to address limitations in existing scoring systems and improve risk stratification in ED settings.

## Supplementary Information

Below is the link to the electronic supplementary material.

Supplementary Material 1

## Data Availability

The data underlying this article are owned by the Chang Gung Medical Foundation. These data cannot be shared publicly due to patient privacy regulations. Access to the data can be obtained upon approval from the Institutional Review Board of the Chang Gung Medical Foundation.
