# Supplementary material for: Early prediction of in-hospital deterioration after emergency department admission using machine learning models
Source: BMC Emerg Med. 2026 Jan 5;26:39. doi: 10.1186/s12873-025-01464-w (PMC12870753; doi:10.1186/s12873-025-01464-w)
Supplement: Supplementary file 1 — Supplementary Material 1 [file 12873_2025_1464_MOESM1_ESM.docx]

Supplemental table 1. Baseline characteristics of patients with and without adverse events.

|  | Without adverse event  n= 219,400 | With adverse event  n= 4,922 | p-value |
| --- | --- | --- | --- |
| Age, year-old, mean$\boldsymbol{\pm}$SD | 66.7 ± 16.4 | 71.3 ± 14.5 | <0.001 |
| Male, n (%) | 125610 (57.3%) | 2900 (58.9%) | 0.02 |
| Body mass index | 23.7 ± 3.7 | 23.5 ± 4.5 | <0.001 |
| ED LOS, minutes | 1676.4 ± 1625.9 | 1695.0 ± 1567.8 | 0.427 |
| Vital sign, mean$\boldsymbol{\pm}$SD |  |  |  |
| BT_a, °C | 36.9 ± 1.1 | 37.0 ± 1.1 | 0.025 |
| HR_a, bpm | 95.7 ± 21.5 | 101.8 ± 23.5 | <0.001 |
| SBP_a, mmHg | 139.5 ± 32.1 | 136.4 ± 33.3 | <0.001 |
| DBP_a, mmHg | 80.2 ± 17.5 | 79.1 ± 18.6 | <0.001 |
| RR_a | 19.7 ± 3.1 | 22.2 ± 4.7 | <0.001 |
| SI_a | 0.7 ± 0.3 | 0.8 ± 0.3 | <0.001 |
| GCSE_a | 3.9 ± 0.5 | 3.7 ± 0.8 | <0.001 |
| GCSV_a | 4.5 ± 1.2 | 3.9 ± 1.7 | <0.001 |
| GCSM_a | 5.8 ± 0.8 | 5.4 ± 1.2 | <0.001 |
| BT_e, °C | 36.4 ± 0.7 | 36.5 ± 0.8 | <0.001 |
| HR_e, bpm | 84.7 ± 17.1 | 95.0 ± 20.7 | <0.001 |
| SBP_e, mmHg | 130.0 ± 28.0 | 121.2 ± 32.1 | <0.001 |
| DBP_e, mmHg | 78.4 ± 15.1 | 76.5 ± 17.0 | <0.001 |
| RR_e | 19.0 ± 2.7 | 22.0 ± 4.8 | <0.001 |
| SI_e | 0.7 ± 0.2 | 0.9± 0.4 | <0.001 |
| GCSE_e | 3.9 ± 0.4 | 3.6 ± 0.8 | <0.001 |
| GCSV_e | 4.5 ± 1.1 | 3.7 ± 1.6 | <0.001 |
| GCSM_e | 5.8 ± 0.7 | 5.3 ± 1.3 | <0.001 |
| Laboratory test |  |  |  |
| White blood cell | 10.4 ± 9.2 | 12.2 ± 13.1 | <0.001 |
| Segment neutrophil, % | 74.7 ± 13.4 | 78.2 ± 12.9 | <0.001 |
| Band neutrophil, % | 0.4 ± 1.8 | 0.9 ± 3.0 | <0.001 |
| Hemoglobin | 11.6 ± 2.4 | 11.4 ± 2.5 | <0.001 |
| Platelet | 218.3 ± 104.2 | 212.9 ± 103.9 | <0.001 |
| Sugar | 156.5 ± 84.8 | 167.4 ± 93.8 | <0.001 |
| Creatinine | 1.6 ± 2.1 | 1.8 ± 2.0 | <0.001 |
| AST | 49.5 ± 164.4 | 63.2 ± 273.3 | <0.001 |
| ALT | 42.2 ± 139.7 | 47.7 ± 189.1 | 0.007 |
| Total bilirubin | 1.4 ± 2.2 | 1.4 ± 2.4 | 0.458 |
| Sodium | 135.4 ± 5.2 | 135.2 ± 6.7 | 0.001 |
| Potassium | 3.9 ± 0.6 | 4.0 ± 0.7 | <0.001 |
| C-Reactive Protein | 47.6 ± 46.5 | 49.9 ± 49.3 | <0.001 |
| Albumin | 3.4 ± 0.3 | 3.4 ± 0.3 | <0.001 |
| Troponin I | 0.1 ± 1.6 | 0.2 ± 2.8 | <0.001 |
| Blood pH | 7.4 ± 0.0 | 7.4 ± 0.1 | <0.001 |
| PCO_2_ | 36.9 ± 3.1 | 39.4 ± 10.4 | <0.001 |
| PO_2_ | 64.3 ± 15.9 | 72.3 ± 32.3 | <0.001 |
| HCO_3_ | 23.4 ± 1.8 | 24.2 ± 4.6 | <0.001 |
| Underlying Medical History, N (%) |  |  |  |
| Hypertension | 95699 (43.6%) | 2229 (45.3%) | 0.02 |
| Diabetes mellitus | 70448 (32.1%) | 1640 (33.3%) | 0.075 |
| Liver cirrhosis | 23755 (10.8%) | 420 (8.5%) | <0.001 |
| Old stroke | 38274 (17.4%) | 849 (17.2%) | 0.735 |
| Heart failure | 26423 (12.0%) | 1116 (22.7%) | <0.001 |
| Coronary artery disease | 29630 (13.5%) | 883 (17.9%) | <0.001 |
| End stage renal disease | 50511 (23.0%) | 1244 (25.3%) | <0.001 |
| Malignancy | 62505 (28.5%) | 1437 (29.2%) | 0.285 |
| ED management |  |  |  |
| Oxygen support | 77812 (35.5%) | 3210 (65.2%) | <0.001 |
| High-flow oxygen support | 4772 (2.2%) | 837 (17.0%) | <0.001 |
| Fluid challenge | 28778 (13.1%) | 1082 (22.0%) | <0.001 |
| Inotropic | 3327 (1.5%) | 276 (5.6%) | <0.001 |

AST: Aspartate Aminotransferase, ALT: Alanine Aminotransferase,

Supplemental table 2. Percentage of missing values by variable across different dataset partitions

|  | Training set  n= 135,806 | Validation set  n= 17,068 | Internal test set  n= 16,933 | External test set  n= 54,515 |
| --- | --- | --- | --- | --- |
| White blood cell | 4389 (3.2%) | 557 (3.3%) | 510(3.0%) | 976 (1.8%) |
| Segment neutrophil, % | 8534 (6.3%) | 1078 (6.3%) | 1058 (6.2%) | 1945 (3.6%) |
| Band neutrophil, % | 8534 (6.3%) | 1078 (6.3%) | 1058 (6.2%) | 1945 (3.6%) |
| Hemoglobin | 7536 (5.5%) | 952 (5.6%) | 967 (5.7%) | 2829 (5.2%) |
| Platelet | 8539 (6.3%) | 1072 (6.3%) | 1089 (6.4%) | 3051 (5.6%) |
| Sugar | 17608 (13.0%) | 2209 (12.9%) | 2175 (12.8%) | 3405 (6.2%) |
| Creatinine | 13893 (10.2%) | 1774 (10.4%) | 1706 (10.1%) | 7396 (13.6%) |
| AST | 94924 (69.9%) | 11929 (69.9%) | 11810 (69.7%) | 49906 (91.5%) |
| ALT | 40098 (29.5%) | 4978 (29.2%) | 5024 (29.7%) | 5665 (10.4%) |
| Total bilirubin | 99664 (73.4%) | 12542 (73.5%) | 12471 (73.6%) | 34676 (63.4%) |
| Sodium | 10843 (8.0%) | 1069 (8.0%) | 1094 (8.2%) | 2947 (5.4%) |
| Potassium | 10416 (7.7%) | 1309 (7.7%) | 1394 (8.2%) | 2862 (5.2%) |
| C-Reactive Protein | 52491 (38.7%) | 6682 (39.1%) | 6579 (38.9%) | 26281 (48.2%) |
| Albumin | 115936 (85.4%) | 14596 (85.5%) | 14427 (85.2%) | 44507 (81.6%) |
| Troponin I | 94586 (69.6%) | 11848 (69.4%) | 11815 (69.8%) | 20900 (38.3%) |
| Blood pH | 117045 (86.2%) | 14663 (85.9%) | 14706 (86.8%) | 54218 (99.5%) |
| PCO_2_ | 117045 (86.2%) | 14663 (85.9%) | 14706 (86.8%) | 54218 (99.5%) |
| PO_2_ | 117045 (86.2%) | 14663 (85.9%) | 14706 (86.8%) | 54218 (99.5%) |
| HCO_3_ | 117045 (86.2%) | 14663 (85.9%) | 14706 (86.8%) | 54218 (99.5%) |

Supplemental table 3. Subgroup analysis of XGBoost model Performance in Linkou and Kaohsiung CGMH cohorts

| Category | AUC | 95% CI Lower | 95% CI Upper |
| --- | --- | --- | --- |
| Age ≥65 years | 0.865119 | 0.849562 | 0.88042 |
| Age <65 years | 0.878035 | 0.857882 | 0.899782 |
| Male sex | 0.863931 | 0.847963 | 0.880009 |
| Female sex | 0.882283 | 0.863161 | 0.901578 |
| Hypertension | 0.878826 | 0.860825 | 0.896617 |
| Without hypertension | 0.8664 | 0.850429 | 0.881809 |
| Diabetes | 0.878353 | 0.85475 | 0.898476 |
| Without diabetes | 0.869324 | 0.855259 | 0.883136 |
| Liver cirrhosis | 0.826789 | 0.777702 | 0.87301 |
| Without liver cirrhosis | 0.875094 | 0.862504 | 0.888264 |
| CVA | 0.85091 | 0.822084 | 0.876659 |
| Without CVA | 0.875871 | 0.861959 | 0.889189 |
| Heart failure | 0.823967 | 0.78938 | 0.857126 |
| Without heart failure | 0.875305 | 0.861074 | 0.888142 |
| CAD | 0.845156 | 0.807862 | 0.881082 |
| Without CAD | 0.875135 | 0.861575 | 0.888984 |
| ESRD | 0.861428 | 0.835843 | 0.885821 |
| Without ESRD | 0.874191 | 0.860592 | 0.88859 |
| Malignancy | 0.854524 | 0.828018 | 0.880959 |
| Without malignancy | 0.877386 | 0.86452 | 0.891451 |
| Overall | 0.870933 | 0.858268 | 0.882952 |

Supplemental table 4. Subgroup analysis of XGBoost model performance in Chiayi CGMH cohort

| Category | AUC | 95% CI Lower | 95% CI Upper |
| --- | --- | --- | --- |
| Age ≥65 years | 0.825017 | 0.807552 | 0.842272 |
| Age <65 years | 0.862055 | 0.837212 | 0.884875 |
| Male sex | 0.822596 | 0.804007 | 0.840628 |
| Female sex | 0.858654 | 0.836054 | 0.880591 |
| Hypertension | 0.83372 | 0.812437 | 0.853591 |
| Without hypertension | 0.841261 | 0.821723 | 0.860089 |
| Diabetes | 0.836032 | 0.813839 | 0.859103 |
| Without diabetes | 0.837168 | 0.819354 | 0.854888 |
| Liver cirrhosis | 0.803083 | 0.747771 | 0.852816 |
| Without liver cirrhosis | 0.840623 | 0.826084 | 0.854069 |
| CVA | 0.822942 | 0.781289 | 0.858311 |
| Without CVA | 0.839938 | 0.82384 | 0.854777 |
| Heart failure | 0.802791 | 0.766568 | 0.835578 |
| Without heart failure | 0.837641 | 0.821071 | 0.852884 |
| CAD | 0.822273 | 0.791231 | 0.851737 |
| Without CAD | 0.836895 | 0.820347 | 0.85398 |
| ESRD | 0.818079 | 0.788001 | 0.846348 |
| Without ESRD | 0.842039 | 0.825635 | 0.857853 |
| Malignancy | 0.798143 | 0.767011 | 0.828795 |
| Without malignancy | 0.85381 | 0.837128 | 0.869553 |
| Overall | 0.836927 | 0.821292 | 0.851189 |
